# Clinical Outcomes of Combined Implantation of an Extended Depth of Focus IOL and a Trifocal IOL in a Korean Population

**DOI:** 10.1155/2021/9034258

**Published:** 2021-09-07

**Authors:** Ji Eun Song, So Young Han, Ramin Khoramnia, Tamer Tandogan, Gerd U. Auffarth, Chul Young Choi

**Affiliations:** ^1^Department of Ophthalmology, Kangbuk Samsung Hospital, Sungkyunkwan University School of Medicine, Seoul, Republic of Korea; ^2^The David J. Apple International Laboratory for Ocular Pathology and International Vision Correction Research Centre (IVCRC), Department of Ophthalmology, University of Heidelberg, Heidelberg, Germany

## Abstract

**Purpose:**

To evaluate monocular and binocular visual performance and patient-reported outcomes following combined implantation of a diffractive extended depth of focus (EDoF) IOL (Carl Zeiss AT LARA 829MP) and a diffractive trifocal IOL (Carl Zeiss AT LISA tri 839MP).

**Methods:**

This prospective study enrolled consecutive patients undergoing lens phacoemulsification of cataract and combined implantation of an EDoF IOL in the dominant eye and a trifocal IOL in the nondominant eye. Assessment included uncorrected visual acuity at near distances (UNVA), intermediate distances (UIVA), and far distances (UDVA), uncorrected defocus curve, contrast sensitivity (CS), reading speed, and patient satisfaction, evaluated six months after the surgery with the Visual Function Questionnaire (VFQ-25).

**Results:**

A total of 25 patients were enrolled. At six months postoperatively, outcomes of binocular UNVA, UIVA, and UDVA were superior to those of monocular outcomes. The binocular defocus curve showed significantly better results in comparison with the AT LISA tri IOL eyes at defocus levels of −1.0 D and −1.5 D (*P*=0.008 and *P*=0.002, respectively) and compared to the AT LARA IOL eyes at defocus levels of −3.0, −3.5 D, and −4.0 D (*P*=0.019, *P*=0.019, and *P*=0.035, respectively). All of the patients were spectacle-free at far and intermediate distances, while 4% of patients needed spectacles at the near distance. Reading speed showed a rather high and gentle slope curve between 0.1 logMAR and 0.4 logMAR, and optical phenomena were improved after combined implantation of IOLs except halos. There were no significant differences in CS between the binocular and monocular results of each IOL.

**Conclusions:**

The combined implantation of an EDoF IOL and a trifocal IOL seems to be a good option for patients with demands for spectacle independence in their daily life, with minimal photic phenomena.

## 1. Introduction

With the advancements in intraocular lenses (IOL) and cataract surgery techniques, it has become increasingly important to minimize visual side effects while improving visual acuity. Traditional cataract surgery with monofocal IOLs can provide excellent uncorrected distance visual acuity outcomes, while spectacle correction is needed for tasks at near and intermediate distances. However, the increasing use of laptops, tablets, and smart phones has made intermediate and near-distance vision important for most patients' daily lives. In recent years, several types of presbyopia-correcting IOLs have been designed, among which trifocal IOLs and extended depth of focus (EDoF) are two mainstream options [1–4]. There are many studies which compare the clinical performance of these presbyopia-correcting IOLs. Trifocal IOLs have almost completely substituted bifocal IOLs because the addition of a third focus can provide better uncorrected visual acuity results at intermediate distances [1, 3, 5, 6]. However, it has been reported that such IOLs may reduce contrast sensitivity and increase visual side effects such as glare and halos because the incoming light energy is split and directed to multiple focal points. For these reasons, in patients with corneal pathologies or other ocular abnormalities, trifocal IOL implantation would lead to dissatisfied patients after surgery, so monofocal IOL implantation may be better in these cases [7, 8]. EDoF IOLs, on the other hand, have been designed to elongate the focal point in order to provide continuous vision from far to near distances without compromising qualitative and quantitative vision [9]. However, worse results for near visual acuity may be achieved in comparison with bifocal or trifocal IOLs [10–12]. A combination of presbyopia-correcting IOLs with different designs is one of the ways to compensate for these limitations and to further enhance results at intermediate and near distances [13–15]. Such a combination in a blended approach has become a topic of interest. This mix-and-match approach has previously been shown to increase visual acuity results while decreasing unwanted photic phenomena [16].

The purpose of this study was to evaluate the monocular and binocular visual performance, contrast sensitivity, reading speed, and patient satisfaction in patients with combined implantation of an EDOF IOL in the dominant eye and a trifocal IOL in the nondominant eye.

## 2. Patients and Methods

This prospective study included patients with age-related cataract who underwent bilateral cataract extraction with phacoemulsification and blended IOL implantation of a diffractive EDoF IOL (AT LARA 829MP, Carl Zeiss, Germany) and a diffractive trifocal IOL (AT LISA tri 839MP, Carl Zeiss, Germany). The study comprised 50 eyes of 25 patients with blended implantation of an AT LARA 829MP in the dominant eye and an AT LISA tri 839MP in the nondominant eye.  Table 1 provides the preoperative patient characteristics. All patients were 21 years or older at the time of enrollment and underwent surgery of the second eye within seven days after surgery of the first eye. The exclusion criteria were the same as previous studies [17]. This study was approved by the Institutional Review Board of Kangbuk Samsung Hospital (IRB File No. 2019-12-039–002), and the tenets of the Declaration of Helsinki were followed. All participants gave their informed consent before enrollment.

### 2.1. Preoperative Assessment

Before surgery, all patients received a complete ophthalmological examination, including uncorrected and corrected visual acuities at far distance (UDVA, CDVA), uncorrected visual acuity at intermediate (UIVA at 66 cm) and near (UNVA at 40 cm) distances, refractive status, mesopic (3 cd/m^2^) pupillometry, topography (Galilei G6; Ziemer Ophthalmic Systems AG, Port, Switzerland), corneal aberration (KR-1W wavefront analyzer, Topcon Europe Medical B. V., Netherlands), optical biometry and keratometry (IOLMaster 700, Carl Zeiss Meditec, Oberkochen, Germany), slit lamp examination, and fundoscopy.

### 2.2. Surgical Technique

One surgeon (CYC) performed the surgeries using topical anesthesia. Phacoemulsification with a 2.2 mm temporal corneal incision and manual capsulorhexis was performed in all cases. All IOLs were implanted in the bag. Postoperative refraction was targeted at the minus value closest to zero using the Barrett True-K formula and Haigis formula for IOL power calculation.

### 2.3. Postoperative Assessment

Follow-up examinations were performed 1 week, 1 month, 3 months, and 6 months after implantation of the second IOL. Main outcome measures included visual performance, monocular and binocular defocus curves, contrast sensitivity (CS), reading speed, and a patient questionnaire. UDVA, UIVA at 66 cm, and UNVA at 40 cm were measured using the Early Treatment Diabetic Retinopathy Study charts (ETDRS; Vector Vision, Ltd., Greenville, OH, USA). Uncorrected monocular and binocular defocus curves were obtained for distance vision with the ETDRS charts at intervals of 0.50 spherical diopters from −4.00 to +1.00 D. CS was measured at 3.0, 6.0, 12.0, and 18.0 cycles per degree (cpd) under photopic (85 cd/m^2^) and mesopic (3cd/m^2^) conditions with and without glare with the CSV-1000 (Vector vision, Inc., Greenville, OH, USA). Patients' subjective satisfaction (quality of vision (QoV) and vision-related quality of life (QoL)) and spectacle independence were assessed with the 25-item National Eye Institute Functional Questionnaire (NEI VFQ-25). Binocular reading speed at 40 cm was measured 6 months postoperatively as described by the Korean Reading Speed Application tester introduced by Kim et al. [18] and using the application of Song et al. [19]. Letter sizes from 0.0 logMAR to 1.0 logMAR were displayed in steps of 0.1 logMAR. Patients were asked to read sentences of different sizes one after the other. Reading speed (words per minute) was automatically calculated by the system. All preoperative and postoperative evaluations were conducted similarly to previous studies [17].

### 2.4. Statistical Analysis

Data analysis was conducted using SPSS (Version 24.0, SPSS Inc., Chicago, IL, USA). Intragroup and intergroup comparisons of monocular and binocular visual outcomes were performed using the Wilcoxon signed-rank test and chi-square test. The Mann–Whitney test was used to compare quantitative variables (such as refraction) and reading speed. Spearman's rank correlation and Pearson's correlation were used to investigate correlations of photopsia. The Student's *t*-test for independent samples was used to compare overall satisfaction and spectacle independence. For the adjustment of *P* values, the Bonferroni correction was used. Data were expressed as means and standard deviations. For all analyses, the level of significance was a *P* value of less than 0.05.

## 3. Results

**Figure 1:**
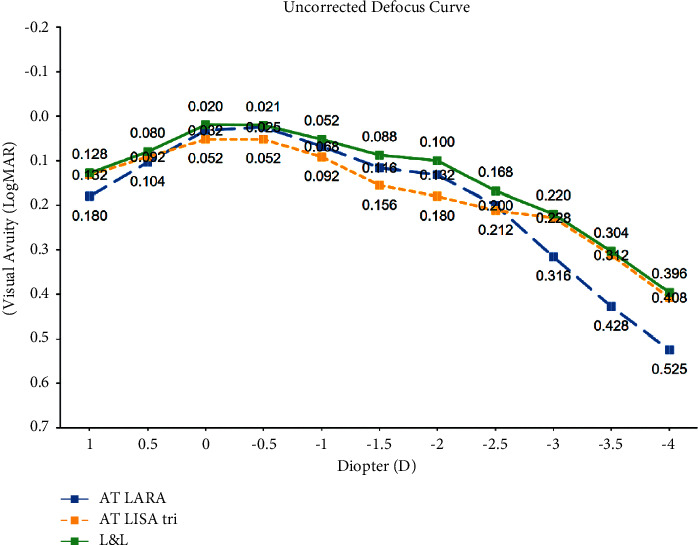
Mean monocular and binocular defocus curves of the eyes implanted with the AT LARA 829MP IOL in the dominant eye and the AT LISA tri 839MP IOL in the nondominant eye.

The mean postoperative UDVA, UIVA, UNVA, CDVA, and refraction are given in Table 2. There were no statistically significant differences between lenses in postoperative uncorrected visual acuity at all distances or in CDVA (*P* > 0.05). The eyes with the AT LARA 829MP achieved a better monocular UIVA compared to the eyes with the AT LISA tri 839MP (*P*=0.09), while the eyes with the AT LISA tri showed a better monocular UNVA compared to the eyes with the AT LARA (*P*=0.59). Although not statistically significant, binocular visual acuities at all distances were better in patients with combined IOL implantation in comparison with monocular visual acuity results achieved with each IOL. A binocular UDVA and UIVA of 0.1 logMAR or better was achieved by 100% of patients with a combined implantation of AT LARA and AT LISA tri. In addition, 100% of patients showed a binocular UIVA 0.2 logMAR or better. Although the spherical equivalent was significantly skewed toward myopic values in the eyes with AT LARA 829MP IOLs compared to the eyes with AT LISA tri 839MP IOLs (*P* < 0.05), the eyes with the AT LISA tri 839MP IOLs showed better visual acuity results in the defocus curve from −3 D to −4 D.  Figure 1 shows the mean monocular and binocular defocus curves. Regarding distance vision (at a vergence of 0.0 D), monocular visual acuity results with both IOLs were similar to the binocular visual acuity outcomes (*P*=0.485, the eyes with the AT LARA; *P*=0.154, the eyes with the AT LISA tri). At an intermediate distance, the binocular defocus curve showed significantly better visual acuity outcomes than the monocular defocus curve in AT LISA tri 839MP IOL-implanted eyes (*P* < 0.05 at −1.5 D and −2.0 D, respectively), and binocular visual acuity results in the near distance were significantly better than monocular outcomes in the eyes implanted with the AT LARA 829MP IOL (*P* < 0.05 at −3 D, −3.5 D, and −4 D, respectively). Overall, the combined implantation of the AT LARA IOL and the AT LISA tri IOL demonstrated better visual acuity results at all distances compared to the monocular results of both IOLs implanted.

**Figure 2:**
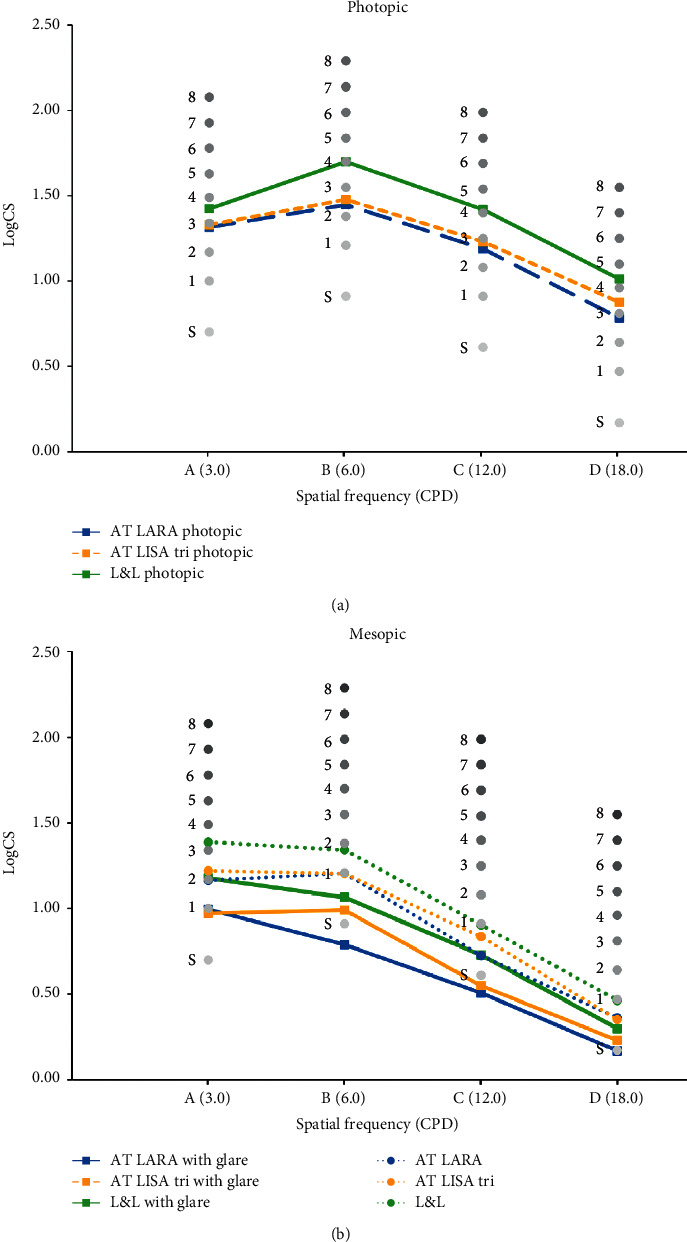
Mean monocular and binocular contrast sensitivity functions under photopic conditions (a) and under mesopic conditions with and without glare (b).

**Figure 3:**
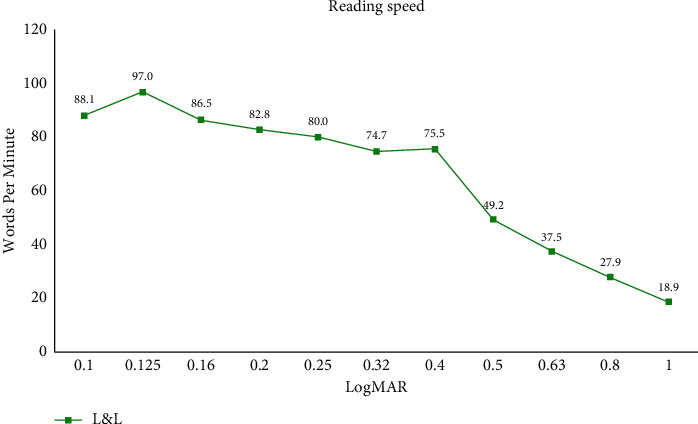
Results of postoperative reading speed at the 6-month follow-up (words per minute).

Figure 2 demonstrates the results of postoperative monocular and binocular CS measurements obtained under mesopic conditions with and without glare and photopic conditions. There were no statistically significant differences for any spatial frequency and light conditions between the two IOLs or between the monocular and binocular outcomes.  Figure 3 shows the binocular reading speed at 40 cm. It shows a rather high and gentle slope curve with a smooth decrease from 0.1 logMAR to 0.4 logMAR, but for smaller letters, the decreasing slope of the reading speed is more pronounced.

**Figure 4:**
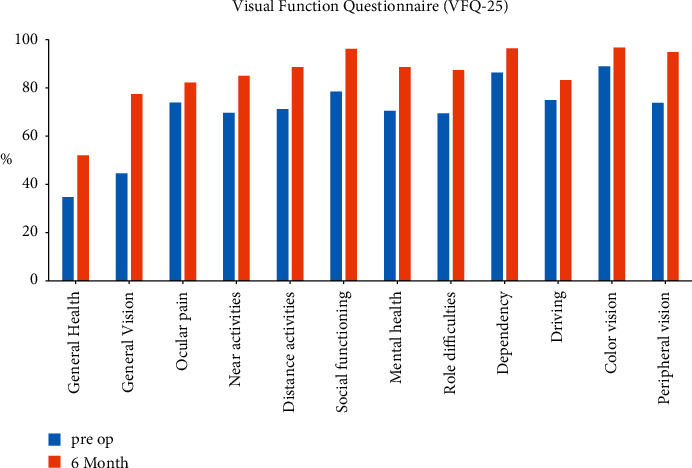
Preoperative and 6-month postoperative results of the VFQ-25.

**Figure 5:**
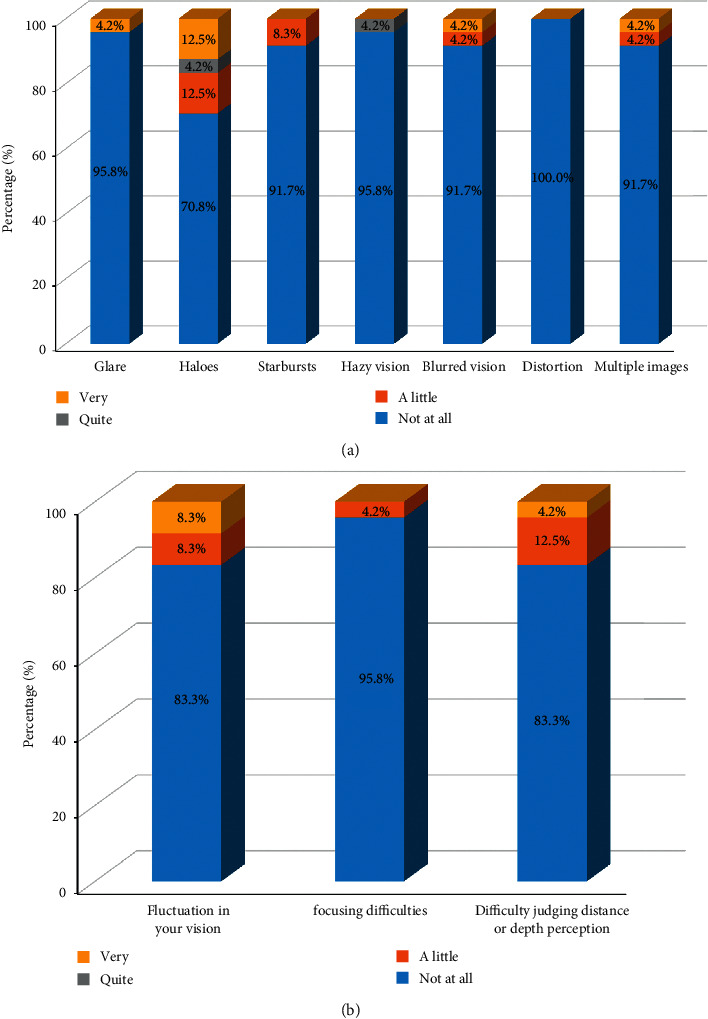
Patient-reported outcomes regarding the perception of optical phenomena.

**Figure 6:**
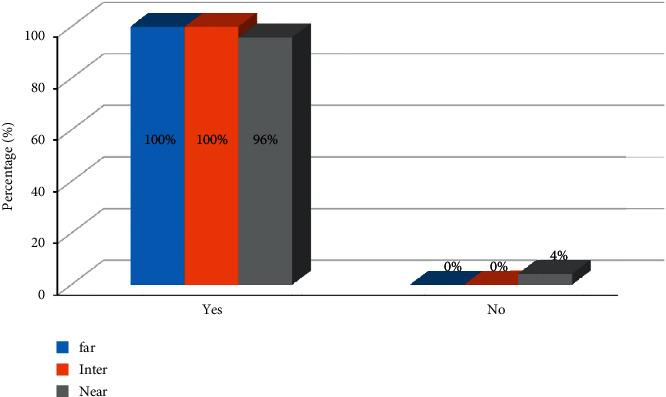
Results of the questionnaire for spectacle independence in daily life for near, intermediate, and far distances.

The postoperative results of the VFQ-25 are shown in Figure 4. Compared to preoperative values, all participants responded with improved outcomes in almost all categories except for ocular pain. Patient-reported postoperative visual phenomena are presented in Figure 5. A noticeable increase in postoperative perception of halos was noted. The proportion of patients bothered by halos rose from 8% before surgery to 29.2% after surgery. A postoperative improvement regarding all the other questions on visual phenomena was noticed. The results of the questionnaire evaluating spectacle independence in daily life are presented in Figure 6. All patients could experience clear vision at the far and intermediate distances, while only 4% of patients needed spectacles at the near distance.

## 4. Discussion

In this study, we implanted the AT LARA 829M in the patients' dominant eye and the AT LISA tri 839MP in the nondominant eye. Visual outcomes of patients with combined IOL implantation demonstrated improved visual acuities at far, intermediate, and even near distances with minimal photic phenomena except for halos.

With the proven benefit of EDoF IOLs with regard to refractive tolerance, improved visual acuity from distance to near is provided, while undesirable visual phenomena are reduced [4, 20]. Although visual acuity at all distances has been improved compared to monofocal IOLs, it has already been shown that bilateral implantation of EDoF IOLs provides inferior visual acuity results at the near distance compared to the results achieved with other types of presbyopia-correcting IOLs [11, 21, 22]. Recently, the blended implantation strategy has been attempted to take advantage of the merits of both IOL types, and good results have been reported [13, 23, 24]. In a previous study, we compared the visual performance of patients with mix-and-match implantation of an EDoF IOL in the dominant eye and a bifocal IOL in the nondominant eye with trifocal IOL implantation in both the eyes [17]. According to this study, patients with the mix-and-match implantation showed better visual acuity results from the far to intermediate distance, while patients with the trifocal IOL achieved better visual acuity results at the near distance. Other studies confirmed the advantages of trifocal IOLs [25]. De Carneros-Llorente et al. reported that trifocal IOLs provide better results at the intermediate distance in comparison with bifocal IOLs without compromising near or distance visual acuity [26]. Since trifocal IOLs can provide a wide range of vision including intermediate distance vision due to the additional focal point, we attempted to perform a combination approach. It was speculated that trifocal IOLs might compensate for the worse visual acuity results of EDoF IOLs at near distances. According to the visual outcomes reported in this study, this assumption turned out to be correct.

In this study, the uncorrected defocus curve was measured to assess the results in real-life conditions. However, a corrected defocus curve rather shows the inherent characteristics of each IOL. Defocus curves allow ophthalmologists to measure the expected range of vision and understand the visual performance of IOLs in order to counsel their patients correctly. The binocular defocus curve of patients with combined IOL implantation represented a slightly wider curve with a higher plateau from the far to near distance than the monocular defocus curve of each IOL. The binocular defocus curve showed significantly better visual acuity results compared to the AT LISA tri IOL eyes at defocus levels of −1.0 D and −1.5 D (*P*=0.008 and *P*=0.002, respectively) and the AT LARA IOL eyes at defocus levels of −3.0, −3.5 D, and −4.0 D (*P*=0.019, *P*=0.019, and *P*=0.035, respectively). Richard et al. showed a similar defocus curve in patients with combined IOL implantation and also presented better visual acuity results at defocus levels of −1.0 D in patients with bilateral EDoF IOL implantation [27]. However, there were only 5 patients with bilateral EDoF IOLs, and no other information is available. For accurate assessment and a direct comparison, a similar number of patients with bilateral implantation of EDoF IOLs and trifocal IOLs would be required.

Patients in this study successfully achieved a visual improvement after cataract surgery at all distances. Spectacle independence at far and intermediate distances was achieved in all patients, while the rate of spectacle independence was just slightly lower at the near distance (96%). Although there was a small number of patients who still needed spectacles for working in the near distance, a very high degree of spectacle independence was achieved with similar outcomes compared to rates in previous studies with the same IOLs [27].

As the use of laptops and smartphones increases, reading speed measurement becomes a valuable predictor that reflects visual performance in everyday life in terms of near vision function [28]. The most widely known devices for reading speed measurements are the MNREAD Chart and Radner Reading Chart, but unfortunately, there is no Korean version available [29–31]. In this study, we used a Korean reading speed application test which is developed appropriately for the Korean writing system called “Hangul.” Hangul is fundamentally based on an alphabetic principle, and letters are printed in square-like blocks composed of three consonants including first, medial, and final consonants. For this reason, reading Korean might be more sensitive to blurring.

In the present study, we also evaluated patients' experience with optical phenomena such as glare, halos, starbursts, hazy vision, blurred vision, distortion, and double vision to understand patients' satisfaction in their daily life. Based on the results of the QoV questionnaire, the most frequently perceived phenomenon was halos (29.2% of patients were suffering from halos), while other optical phenomena were improved compared to before surgery. Previous studies have reported that the neuroadaptation process may reduce these optical phenomena over time after surgery [32, 33]. The neuroadaptation process after presbyopia-correcting IOL implantation usually involves a minimum of 3 months and can last up to 1 year. In this study, however, the last follow-up was at 6 months, when the neuroadaptation process is still in process. It could be assumed that difficulties related to optical phenomena might decrease over time; thus, further research with a longer follow-up would be needed.

To summarize, the combined implantation of EDoF and trifocal IOLs can improve corrected and uncorrected visual acuities from far to near distances. Spectacle independence was high at all distances. As shown by the defocus curve, patients with combined IOL implantation achieved better visual acuity results at intermediate and near distances without compromising far distance vision compared to the monocular outcomes of each IOL. The combined implantation of an EDoF and a trifocal IOL can be a viable option for patients with high demands for spectacle independence in their daily life with minimal optical phenomena. In addition, it can be used as a background study of relatively safe recommendations other than monovision for patients who complain of each deficiency after the insertion of an EDoF or trifocal IOL in their first eye.

## Figures and Tables

**Table 1:** Preoperative characteristics of patients.

	LARA	LISA tri	*P* value
Age (years)	66.6 ± 6.38		

Gender			
Male	5		
Female	20		

Pupil size (mm)	3.77 ± 1.13	3.65 ± 1.07	0.617

Refraction			
Sph (D)	1.09 ± 1.79	1.19 ± 1.70	0.803
Cyl (D)	−0.83 ± 0.67	−0.83 ± 0.53	0.939
SE (D)	0.68 ± 1.71	0.78 ± 1.72	0.811

Data are expressed as mean ± standard deviation or number. Sph, sphere; Cyl, cylinder; D, diopter; SE, spherical equivalent.

**Table 2:** Monocular and binocular visual outcomes 6 months postoperatively.

	LARA	LISA tri	L&L	*P* value		
				LARA vs. LISA tri	LARA vs. L&L	LISA tri vs. L&L
VA						
UDVA	0.04 ± 0.06	0.04 ± 0.09	0.02 ± 0.05	0.85	0.18	0.23
UIVA	0.04 ± 0.07	0.07 ± 0.10	0.03 ± 0.05	0.09	0.48	0.05
UNVA	0.11 ± 0.12	0.09 ± 0.09	0.07 ± 0.08	0.59	0.17	0.33
CDVA	−0.01 ± 0.06	0.00 ± 0.10	−0.02 ± 0.07	0.74	0.51	0.41

Refraction						
Sph (D)	−0.47 ± 0.44	0.00 ± 0.42	<0.001			
Cyl (D)	−0.63 ± 0.52	−0.81 ± 0.52	0.21			
SE (D)	−0.79 ± 0.36	−0.41 ± 0.29	<0.001			

Data are expressed as mean ± standard deviation (range). VA, visual acuity; UDVA, uncorrected distance visual acuity (logMAR); UIVA, uncorrected intermediate visual acuity (logMAR); UNVA, uncorrected near visual acuity (logMAR); CDVA, corrected distance visual acuity; Sph, sphere; Cyl, cylinder; D, diopter; SE, spherical equivalent.

## Data Availability

The data used to support the findings of this study are available from the corresponding author upon request.

## References

[1] Cochener B., Vryghem J., Rozot P. (2014). Clinical outcomes with a trifocal intraocular lens: a multicenter study. *Journal of Refractive Surgery*.

[2] Jonker S. M. R., Bauer N. J. C., Makhotkina N. Y., Berendschot T. T. J. M., van den Biggelaar F. J. H. M., Nuijts R. M. M. A. (2015). Comparison of a trifocal intraocular lens with a +3.0 D bifocal IOL: results of a prospective randomized clinical trial. *Journal of Cataract & Refractive Surgery*.

[3] Shen Z., Lin Y., Zhu Y., Liu X., Yan J., Yao K. (2017). Clinical comparison of patient outcomes following implantation of trifocal or bifocal intraocular lenses: a systematic review and meta-analysis. *Scientific Reports*.

[4] Pedrotti E., Bruni E., Bonacci E., Badalamenti R., Mastropasqua R., Marchini G. (2016). Comparative analysis of the clinical outcomes with a monofocal and an extended range of vision intraocular lens. *Journal of Refractive Surgery*.

[5] Xu Z., Cao D., Chen X., Wu S., Wang X., Wu Q. (2017). Comparison of clinical performance between trifocal and bifocal intraocular lenses: a meta-analysis. *PloS One*.

[6] Son H. S., Tandogan T., Liebing S. (2017). In vitro optical quality measurements of three intraocular lens models having identical platform. *BMC Ophthalmology*.

[7] Meduri A., Urso M., Signorino G. A., Rechichi M., Mazzotta C., Kaufman S. (2017). Cataract surgery on post radial keratotomy patients. *International Journal of Ophthalmology*.

[8] Braga-Mele R., Chang D., Dewey S. (2014). Multifocal intraocular lenses: relative indications and contraindications for implantation. *Journal of Cataract & Refractive Surgery*.

[9] Schallhorn S. C., Teenan D., Venter J. A., Hannan S. J., Schallhorn J. M. (2019). Initial clinical outcomes of a new extended depth of focus intraocular lens. *Journal of Refractive Surgery*.

[10] Kondylis G., Klavdianou O., Palioura S. (2019). Multifocal and extended depth of focus intraocular lenses. *Annals of Eye Science*.

[11] Cochener B., Boutillier G., Lamard M., Auberger-Zagnoli C. (2018). A comparative evaluation of a new generation of diffractive trifocal and extended depth of focus intraocular lenses. *Journal of Refractive Surgery*.

[12] Black S. (2018). A clinical assessment of visual performance of combining the TECNIS® Symfony Extended Range of Vision IOL (ZXR00) with the +3.25 D TECNIS Multifocal 1-piece IOL (ZLB00) in subjects undergoing bilateral cataract extraction. *Clinical Ophthalmology*.

[13] de Medeiros A. L., de Araujo Rolim A. G., Motta A. F. P. (2017). Comparison of visual outcomes after bilateral implantation of a diffractive trifocal intraocular lens and blended implantation of an extended depth of focus intraocular lens with a diffractive bifocal intraocular lens. *Clinical Ophthalmology*.

[14] Breyer D. R. H., Kaymak H., Ax T., Kretz F. T. A., Auffarth G. U., Hagen P. R. (2017). Multifocal intraocular lenses and extended depth of focus intraocular lenses. *Asia-Pacific journal of ophthalmology (Philadelphia, Pa.)*.

[15] Jacobi F. K., Kammann J., Jacobi K. W., Grosskopf U., Walden K. (1999). Bilateral implantation of asymmetrical diffractive multifocal intraocular lenses. *Archives of Ophthalmology*.

[16] Jiang Y., Bu S., Tian F. (2019). Long-term clinical outcomes after mix and match implantation of two multifocal intraocular lenses with different adds. *Journal of Ophthalmology*.

[17] Song J. E., Khoramnia R., Son H.-S., Knorz M. C., Choi C. Y. (2020). Comparison between bilateral implantation of a trifocal IOL and mix-and-match implantation of a bifocal IOL and an extended depth of focus IOL. *Journal of Refractive Surgery*.

[18] Kim M., Kim J.-h., Lim T.-H., Cho B. J. (2018). Comparison of reading speed after bilateral bifocal and trifocal intraocular lens implantation. *Korean Journal of Ophthalmology*.

[19] Song J., Kim J.-h., Hyung S. (2016). Validity of Korean version reading speed application and measurement of reading speed: pilot study. *Journal of the Korean Ophthalmological Society*.

[20] Pedrotti E., Carones F., Aiello F. (2018). Comparative analysis of visual outcomes with 4 intraocular lenses: monofocal, multifocal, and extended range of vision. *Journal of Cataract & Refractive Surgery*.

[21] Monaco G., Gari M., Di Censo F., Poscia A., Ruggi G., Scialdone A. (2017). Visual performance after bilateral implantation of 2 new presbyopia-correcting intraocular lenses: trifocal versus extended range of vision. *Journal of Cataract & Refractive Surgery*.

[22] Son H.-S., Kim S. H., Auffarth G. U., Choi C. Y. (2019). Prospective comparative study of tolerance to refractive errors after implantation of extended depth of focus and monofocal intraocular lenses with identical aspheric platform in Korean population. *BMC Ophthalmology*.

[23] Martins Cortez Vilar C., Hida W. T., Medeiros A. (2017). Comparison between bilateral implantation of a trifocal intraocular lens and blended implantation of two bifocal intraocular lenses. *Clinical Ophthalmology*.

[24] Gundersen K. G., Potvin R. (2016). Comparison of visual outcomes and subjective visual quality after bilateral implantation of a diffractive trifocal intraocular lens and blended implantation of apodized diffractive bifocal intraocular lenses. *Clinical Ophthalmology (Auckland, N.Z.)*.

[25] Akman A., Asena L., Ozturk C., Güngör S. G. (2019). Evaluation of quality of life after implantation of a new trifocal intraocular lens. *Journal of Cataract & Refractive Surgery*.

[26] de Carneros-Llorente A. M., de Carneros A. M., de Carneros-Llorente P. M., Jiménez-Alfaro I. (2019). Comparison of visual quality and subjective outcomes among 3 trifocal intraocular lenses and 1 bifocal intraocular lens. *Journal of Cataract & Refractive Surgery*.

[27] McNeely R. N., Moutari S., Palme C., Moore J. E. (2020). Visual outcomes and subjective experience after combined implantation of extended depth of focus and trifocal IOLs. *Journal of Refractive Surgery*.

[28] Chia E.-M., Wang J. J., Rochtchina E., Smith W., Cumming R. R., Mitchell P. (2004). Impact of bilateral visual impairment on health-related quality of life: the Blue Mountains Eye Study. *Investigative Opthalmology & Visual Science*.

[29] Legge G. E., Ross J. A., Isenberg L. M., Lamay J. M. (1992). Psychophysics of reading. XII. Clinical predictors of low-vision reading speed. *Investigative Ophthalmology & Visual Science*.

[30] Legge G. E., Ross J. A., Luebker A., LaMay J. M. (1989). Psychophysics of reading. VIII. The Minnesota low- vision reading test. *Optometry and Vision Science*.

[31] Radner W., Obermayer W., Richter-Mueksch S., Willinger U., Velikay-Parel M., Eisenwort B. (2002). The validity and reliability of short German sentences for measuring reading speed. *Graefe’s Archive for Clinical and Experimental Ophthalmology*.

[32] Alió J. L., Pikkel J. (2019). Multifocal intraocular lenses: neuroadaptation. *Essentials in Ophthalmology*.

[33] Alió J. L., Pikkel J. (2019). *Multifocal Intraocular Lenses: The Art and the Practice*.

